# Effects of enriched environment on the expression of β-amyloid and transport-related proteins LRP1 and RAGE in chronic sleep-deprived mice

**DOI:** 10.1515/tnsci-2022-0301

**Published:** 2023-09-01

**Authors:** Ren Yuan, Zhang Yisen, Wang Xiu, Tang Wei, Wang Wei

**Affiliations:** Department of Neurology, Affiliated Xinhua Hospital of Dalian University, Dalian, Liaoning Province, China; Department of Clinical Laboratory, Wuhan Children’s Hospital Affiliated to Tongji Medical College, Huazhong University of Science and Technology, Hubei Province, China; Department of Basic Medicine, School of Medicine of Dalian University, Dalian, Liaoning Province, China

**Keywords:** enriched environment, chronic sleep deprivation, Aβ, LRP1, RAGE

## Abstract

Sleep plays an important role in the learning process and memory consolidation, and sleep deprivation (SD) leads to inadequate memory consolidation and plays an important role in brain development and plasticity. SD increases β-amyloid levels while impairing cognitive function. We explored the effect of enriched environment (EE) on β-amyloid and transporter protein LRP1 and receptor for advanced glycosylation end-products (RAGE) expression in chronic sleep deprived mice. We randomly divided mice into four groups (*n* = 10), the standard environment group (Ctrl group), the sleep deprivation group (SD group), the enriched environment intervention group (EE group), and the sleep deprivation plus environmental enrichment intervention group (SD + EE group). A modified multi-platform SD model was used to sleep deprive the mice for 19 h per day. Five hours of EE intervention was performed daily in the EE group and the SD + EE group, respectively. The behavioral measurements of mice were performed by Y-maze method and new object recognition; the expression levels of Aβ1-42, LRP1, and RAGE in prefrontal cortex and hippocampus of mice were measured by immunofluorescence; the expression levels of LRP1 and RAGE in prefrontal cortex and hippocampus were detected by Western blot. The results showed that EE could effectively ameliorate the effects of SD on cognitive impairment, reduce SD induced Aβ deposition, and decrease the expression of RAGE, while increase the expression of LRP1.

## 1 Introduction

Sleep is an essential behavior for the survival and integrity of the organism and plays an important role in the homeostasis of the body [1]. An adequate and high-quality sleep helps remove waste from the brain [2] and memory processing [3]. It has a positive impact on memory consolidation and storage, learning, and new synapse formation [4]. In contrast, chronic sleep deficiency can impair brain function and increase the risk of neurodegenerative diseases such as Alzheimer’s disease (AD) [5]. Chronic insomnia has been recognized as a high-risk factor for the development of AD and a major cause of dementia. In China, the prevalence of AD in people over 60 years of age is about 4% [6]. The higher prevalence of dementia in urban areas compared to rural areas is often associated with shorter average sleep duration [7]. Sleep deprivation (SD) [8] is the reduced duration of physiologically necessary sleep due to various physiological, pathological, or environmental factors. SD leads to an impaired cognitive function, especially memory and spatial learning, and imposes an increased social, economic, and medical burden. However, the exact pathogenic mechanisms associated with this cognitive decline remain elusive, and no ideal treatment is currently available [9]. Therefore, it is crucial to urgently understand the disease pathogenesis and explore effective treatments.

The concept of the enriched environment (EE) was first introduced by the Canadian scholar Donald Hebb in 1947 [10]. With the development of research, the definition of EE has been gradually refined. It refers to enriched housing conditions with enhanced sensory, motor, cognitive, and social stimuli compared to the standard environment [11]. Many studies have shown that EE can improve learning memory in various animal models of cognitive dysfunction, such as AD [12], cerebral ischemia-reperfusion [13], stress, depression [14], stroke [15], and traumatic brain injury [16], etc. However, few studies have investigated whether and how EE interventions can improve learning and spatial memory abilities after SD exposure. Therefore, the development of EE shows promising therapeutic potential for SD-induced cognitive decline.

The amyloid-β (Aβ) peptide is generated from amyloid precursor protein (APP) by sequential cleavage of β-secretase and γ-secretase [17]. While the Aβ_1–40_ peptide is its most abundant variant, the Aβ_1–42_ isoform closely associated with AD pathogenesis and memory impairment [18]. Under physiological conditions, after Aβ production in the brain, most extracellular Aβ peptides can be enzymatically transported out of the brain to the periphery, which is a very important clearance pathway. Low-density lipoprotein receptor-related protein 1 (LRP1) is the most important transporter protein that traffics Aβ across the blood–brain barrier (BBB) to the periphery, while the receptor for advanced glycosylation end-products (RAGE) mediates the influx of Aβ from the peripheral circulation into the central nervous system [19]. In peripheral plasma, soluble LRP1 and soluble RAGE bind to Aβ and help Aβ to be cleared by the liver or other peripheral organs [20]. In recent years, several studies have shown that LRP1 and RAGE receptors significantly promote free unbound Aβ between the blood and brain and across the BBB in AD models, which may lead to the clearance or accumulation of Aβ through LRP1 and RAGE-mediated Aβ uptake pathways [21,22,23]. Meanwhile, studies have shown that the expression of LRP1 and RAGE in the prefrontal cortex and hippocampus may affect learning and memory ability [21,24,25,26].

In animal experiments, SD exposure has been shown to cause Aβ deposition as well as to affect the expression levels of LRP1 or RAGE in the endothelium of the BBB [27]. However, it is not clear whether the EE administration has an effect on Aβ clearance by modulating the expression or function of LRP1 and RAGE in the BBB. On this basis, we have kept mice in standard or EE housing condition until 10 months of age with or without a 2-month-long rapid eye movement (REM) SD mouse model. The ameliorating potential of EE on the impacts of SD on cerebral Aβ level and learning/memory behavior was evaluated. Moreover, the expression of LRP1 and RAGE proteins was also determined, so as to reveal the mechanism of EE effects. Our data will provide valuable experimental evidence to explore the benefits of non-pharmacological EE interventions on sleep disorders.

## 2 Materials and methods

### 2.1 Experimental animals and grouping

**Figure 1:**
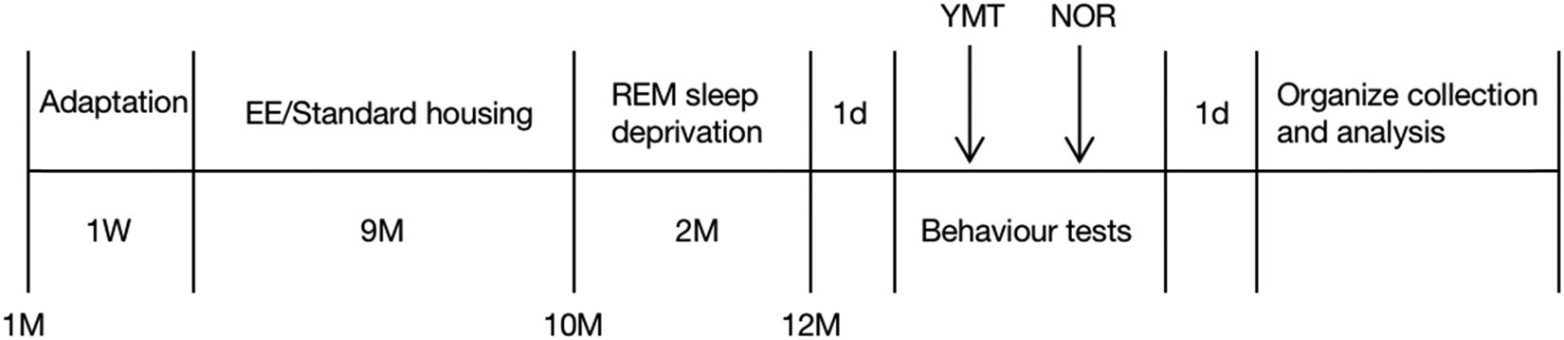
Schematic diagram of the experiment time. Note: EE: enriched environment; YMT: Y-maze test; NOR: new object recognition experiment.

A total of 40 SPF-grade male 1-month-old Kunming mice (21–25 g body weight) were purchased from Liaoning Changsheng Biotechnology Co. The animals were randomized into four groups: the standard environment group (Ctrl), the SD group, the EE group, and the SD + EE group, with 10 animals in each group and 5 animals per cage. The experimental design is schematically illustrated in Figure 1. The mice were housed in the same cage in the animal laboratory for 1 week and, then, the experiment began after adapting to the environment. Sufficient food and drinking water were provided during adapting to the environment, and 12 h (6:00–18:00) light and 12 h (18:00–6:00) dark conditions were alternated.

### 2.2 Animal model making

#### 2.2.1 Housing conditions

The standard environment is standard laboratory cages (32 cm × 17.5 cm × 14.5 cm). For the EE administration, larger cages (54 cm × 33 cm × 20 cm) were used, in which various stimuli were equipped including running wheels, mazes, pipes, small houses, swings, and toys per cage. In the present study, four different types of EE cages were used, which were changed and rearranged weekly to increase the novelty. The Ctrl and SD groups were assigned to standard housing environment, whereas the EE and SD + EE groups were housed in EE condition. All mice were kept in respective housing conditions for up to 9 months till 10 months of age. Mice were housed 4–5 per cage to ensure proper social interactions. In all conditions, food and water were provided *ad libitum*.

#### 2.2.2 REM SD

The modified multiple platform method was used to induce REM SD. The device (39 cm × 27 cm × 20 cm) consisted of a number of small platforms (10 cm high, 3 cm in diameter, 5 cm apart) or large platforms (10 cm high, 10 cm in diameter, 2 cm apart) surrounded by water and located 2 cm above the water surface, on which mice could jump freely and reach for food and water on the top of the tank. The small platform prevented the animals from sleeping because when they fell asleep, they would fall into the water and wake up. After mice were kept in different housing conditions until 10 months of age, mice in the SD and SD + EE groups were placed on the small platform to selectively eliminate REM SD caused by REM sleep. To exclude possible effects of environmental stress, the Ctrl and EE groups were placed in the larger platform that was large enough that the mice would not fall into the water during sleep. The mice were placed in the large and small platforms from 14:00 each day to 9:00 the next morning, with 19 h of SD per day. For the remainder of the day, the mice were moved back to the appropriate standard cage or EE cage for rest. A total of 2 months of SD were used until the mice were 12 months old for the next experiment.

### 2.3 Y-maze test

The Y-maze test is used to assess the function of learning and memory in animals. Mice were placed through the center of the Y-maze and allowed to explore the maze freely for 5 min. An exploration was considered valid when the mouse’s body and tail were completely inside one of the arms, and normal mice were able to enter all three arms sequentially instead of returning to the previous one. All arms were cleaned by using alcohol after completing the test of each mouse to eliminate olfactory interference stimuli. The total number of alternations and sequences of each mouse entering the three arms were recorded via Smart 3.0 software, with the three consecutive arms being considered the correct entry arms (e.g., ACB, CAB). The alternation rate was calculated using the following formula: correct alternation rate = number of correct alternations/(total number of alternations − 2). Also, the total number of arm entries for each mouse was recorded to test the animal’s motility.

### 2.4 Novel object recognition test

Novel object recognition is a behavioral test evaluating the animal’s cognition, particularly recognition memory. The test consists of three sessions: (1) Adaptation session: the mice to be tested were placed in an empty opaque box (50 cm × 50 cm × 60 cm) for 2 days (10 min/each mouse per day) before the experiment started. (2) Familiarization session: three objects A, B, and C were selected, where A and B objects were identical and C was completely different from A and B objects. The two identical objects A and B were placed on the left and right ends of the square box and place the mouse into the box between two objects and facing the side wall, taking care to ensure that the distance from the tip of the mice’s nose to the two objects A and B was the same. The mice were put into the square box for 5 min, and the contacts between the mice and the two objects were recorded using a video camera, and the probing time within 1 cm from the tip of the mice’s nose to the objects was recorded (including the time spent licking the objects, resting the front paws on the objects and sniffing the objects with the nose, climbing onto the objects without moving and posing could not be regarded as probing the new objects). (3) Test session: replace object B in the square box with object C, which is different from B, with the same position and still place the mouse between the two objects with their backs to each other. The mice were observed in the square box for 5 min, and the images captured by the camera were analyzed by Smart 3.0 software (Panlab), and the contact between the mice and the two objects was recorded. The total exploration time and novel object preference of the familiarization and test phases were calculated separately: RI = exploration time of object C/total exploration time, which was used to judge the cognitive function of the mice.

### 2.5 Immunofluorescence (IF) staining

After the behavioral test, the mice were perfused intracardially with PBS, and the brains were removed after full perfusion. The mouse brain tissue was divided into left and right hemispheres with a sharp blade. To exclude the error caused by the difference between the left and right hemispheres, the left hemisphere was fixed with 4% paraformaldehyde for the IF.

#### 2.5.1 Aβ1-42 IF staining

Sections were fixed with 4% paraformaldehyde for half an hour and blocked with 10% goat serum at 37°C, incubated with primary antibodies: rabbit anti-Aβ1–42 (1:200, ab201060; Abcam) overnight at 4°C, and goat anti-rabbit IgG containing fluorescein (Alexa Fluor Plus 594, 1:1,000; Thermo) at room temperature for 1 h. Staining was performed with 2-(4-amidinophenyl)-6-indolemethylamidine dihydrochloride (DAPI). The anti-fluorescence quenching tablet containing Hoechst33342 (P0133, Biyuntian) was used to seal tablets. Photographs were taken using a Pannoramic DESK, P-MIDI (3D HISTECH, Hungary) scanner. The percentage of positive areas was analyzed using ImageJ software.

#### 2.5.2 IF double staining of LRP1, RAGE, and CD31

Sections were fixed using 4% paraformaldehyde and blocked with 10% goat serum at 37°C. The primary antibody was incubated overnight at 4°C, and the secondary antibody was incubated for 1 h at 37°C following the day. TSA was incubated at 37°C for 30 min. After washing with PBS, the fixation step, serum blocking, and antibody incubation were repeated. After final fixation, the cells were stained with DAPI. Anti-fluorescence quencher containing Hoechst33342 (P0133, Biyuntian) was used to seal tablets. The primary antibodies were rabbit anti-LRP1 (1:200, ab92544; Abcam), rabbit anti-RAGE (1:200, ab3611; Abcam), and rabbit anti-CD31 (1:2,000, ab182981; Abcam). The second antibodies were horseradish peroxidase (HRP)-conjugated goat anti-rabbit IgG (1:300, 074-1506, KPL) and HRP-conjugated goat anti-mouse IgG (1:300, 074-1806, KPL). Photographs were taken using a Pannoramic DESK, P-MIDI (3D HISTECH, Hungary) scanner. The percentage of positive areas was analyzed using ImageJ software. ImageJ was used to analyze LRP1-CD31 co-localization area, CD31 expression area, RAGE-CD31 co-localization area, and CD31 expression area in the hippocampus and cortex, respectively, and the ratio of co-localization area to CD31 area represents the relative amount of positive protein expressed per unit endothelial cell.

### 2.6 Western blotting

The mice heart was perfused and after brain tissue was removed, the hippocampus and prefrontal cortex of the right hemisphere were isolated. Hippocampal and prefrontal cortex tissues were ground in Lysis Buffer (KGP250, KGI Bio) containing protease inhibitors and phosphatase inhibitors. After collection of the supernatant, protein samples were quantified using the Bradford Protein Assay Kit (PC0010; Solarbio). A total of 20 µg of each protein sample was used for 10% sodium dodecyl sulfate polyacrylamide gel electrophoresis followed by wet transfer onto PVDF membranes (Millipore). The samples were blocked for 20 min using QuickBlock™ Western blocking solution (P0252; Beyotime), washed 3 times in TBST, and then incubated with primary antibodies overnight at 4°C. The following primary antibodies were used: Rabbit anti-mouse LRP1 primary antibody (ab92544, 1:1,000; Abcam), Rabbit anti-mouse RAGE primary antibody (ab3611, 1:1,000; Abcam), and Rabbit anti-mouse GAPDH primary antibody (AP0063, 1:5,000; Bioworld). GAPDH was used as an internal reference protein. The membranes were washed 3 times in TBST and incubated with the corresponding secondary antibody (TG266717, 1:10,000; Thermo) for 2 h at room temperature. Protein bands were detected using the Ultrasensitive ECL Chemiluminescence Kit (4AW011; Four Massive Park Bio) according to product specifications. The grayscale values of the strips were quantified using ImageJ software.

### 2.7 Statistical analysis

Statistical analysis was performed using SPSS 24.0 software. All data were expressed as mean ± standard deviation. Multiple group comparisons were performed by one-way ANOVA combined with LSD *post hoc* multiple testing, and *P* < 0.05 was considered a statistically significant difference.


**Ethical approval:** The research related to animals’ use has been complied with all the relevant national regulations and institutional policies for the care and use of animals.

## 3 Results

### 3.1 Effects of EE on learning, memory, and cognitive abilities in chronic sleep-deprived mice

**Figure 2:**
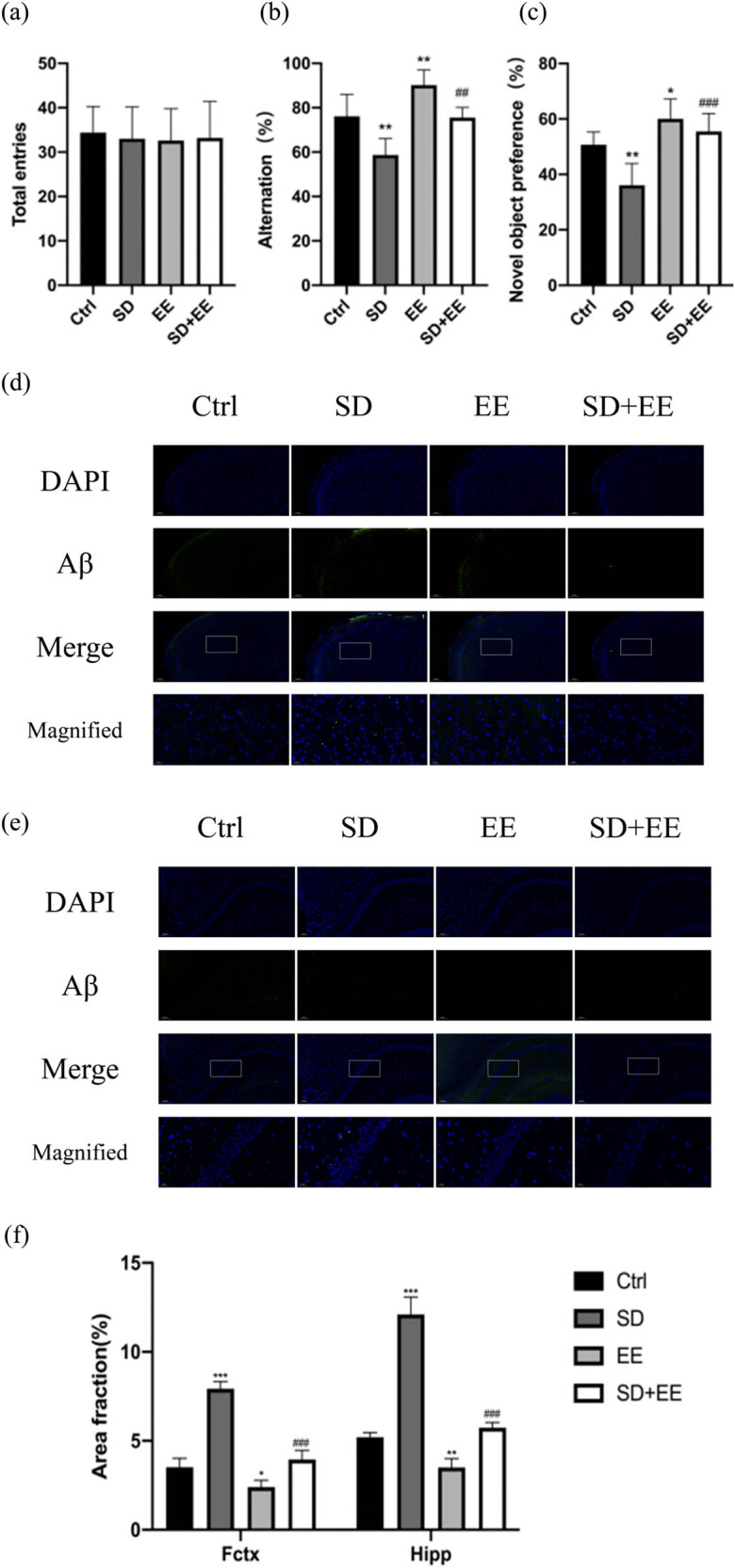
(a–c) Comparison of the results of Y-maze and new object recognition experiments for each group of mice. Note: (a) total number of Y-maze arms; (b) Y-maze alternation rate; (c) new object recognition discrimination index (*n* = 10. *vs Ctrl group, *P < 0.05, **P < 0.01. ^#^vs SD group, ^##^ *P* < 0.01, ^##^ *P* < 0.001); (d) immunofluorescence staining of prefrontal cortex Aβ (green) and DAPI (blue) in each group: magnification scale bar = 100 μm, other scale bar = 20 μm; (e) immunofluorescence staining of prefrontal cortex and hippocampal Aβ (green) and DAPI (blue) in each group: magnification scale bar = 50 μm, other scale bar = 20 μm; and (f) area fraction of positive prefrontal cortex and hippocampus Aβ1-42 in each group (*n* = 3. *P < 0.05, **P < 0.01, ***P < 0.001 compared to the Ctrl group. ^##^ *P* < 0.001 compared to the SD group).

The Y-maze and new object recognition experiments were used to detect the effects of EE on the learning memory ability of sleep-deprived mice. As shown in Figure 2a, the four groups of mice had similar total arm counts and similar mobility, and the differences were not statistically significant (Figure 2b, *P* > 0.05). Compared with the Ctrl group, the alternation rate was decreased in the mice in the SD group (Figure 2b, *P* < 0.01) and increased in the EE group (Figure 2b, *P* < 0.01). Compared with the SD group, the alternation rate was increased in the EE + SD group. (Figure 2b, *P* < 0.01).

The new object recognition experiment showed that compared with the Ctrl group, the discrimination index of the SD group was decreased (Figure 2c, *P* < 0.01), and the discrimination index of the EE group was increased (Figure 2c, *P* < 0.05). Compared with the SD group, the discrimination index of EE + SD group was increased (Figure 2c, *P* < 0.001).

### 3.2 Effects of EE on Aβ1-42 deposition in prefrontal cortex and hippocampus of chronic sleep-deprived mice

IF staining of the prefrontal cortex and hippocampus of the mice was performed separately to assess changes in the expression levels of Aβ1-42 in normal and chronic sleep-deprived mice with and without EE treatment. Our IF results showed that compared with the Ctrl group, the SD group had varying degrees of increased Aβ1-42 in the prefrontal cortex (Figure 2d, *P* < 0.001) and hippocampus (Figure 2e, *P* < 0.001). The expression of Aβ1-42 was decreased in the prefrontal cortex ((Figure 2d, *P* < 0.05) and hippocampus ((Figure 2e, *P* < 0.05) in the EE group. Compared with the SD group, the expression of Aβ1-42 was decreased in the prefrontal cortex (Figure 2d, *P* < 0.001) and hippocampus (Figure 2e, *P* < 0.01) in the SD + EE group.

### 3.3 Effect of EE on LRP1 expression in chronic sleep-deprived mice

**Figure 3:**
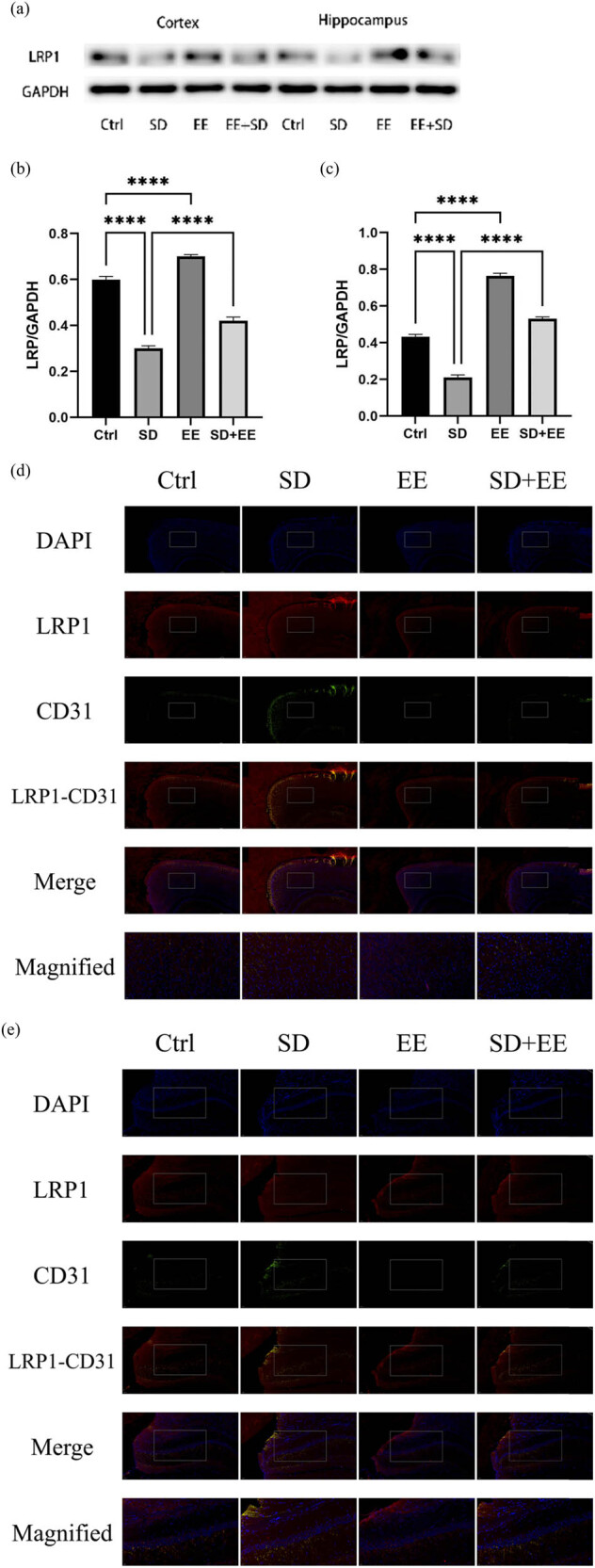

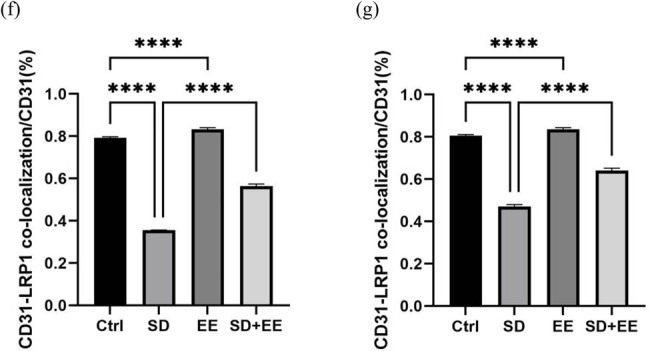
Effect of EE on LRP1 expression in chronic sleep-deprived mice. Note: (a) prefrontal cortex and hippocampal LRP1 protein bands in each group; (b and c) analysis of grayscale values of prefrontal cortex and hippocampal LRP1/GAPDH in each group; (d) immunofluorescence staining of prefrontal cortex LRP1 (red), CD31 (green), and DAPI (blue) in each group: magnification scale bar = 100 μm, other scale bar = 20 μm; (e) immunofluorescence staining of prefrontal cortex and hippocampal LRP1 (red), CD31 (green), and DAPI (blue) in each group: magnification scale bar = 50 μm, other scale bar = 20 μm; (f) percentage of CD31-LRP1 co-localization area of prefrontal cortex stretching CD31 area in each group; and (g) percentage of CD31-LRP1 co-localization area of hippocampal stretching CD31 area in each group (*n* = 3, ****P < 0.001, *P < 0.01).

To assess the effect of the EE administration on LRP1 and RAGE, the expression levels of these two proteins in the prefrontal cortex and hippocampus of mice in each group were determined by Western blot and IF staining. LRP1 is widely expressed in the BBB endothelium, and one of its functions is to transport Aβ from brain tissue to the blood circulation, thus exerting a role in cleaning Aβ from the brain [28]. The Western blot results of LRP1 showed that compared with the Ctrl group, the expression of LRP1 protein was decreased in the prefrontal cortex (Figure 3a and b, *P* < 0.001) and hippocampus (Figure 3a and c, *P* < 0.001) in the SD group, and in the prefrontal cortex (Figure 3a and b, *P* < 0.001) and hippocampus (Figure 3a and c, *P* < 0.001) in the EE group. Compared with the SD group, LRP1 protein expression was decreased in the prefrontal cortex (Figure 3a and b, *P* < 0.001) and hippocampus (Figure 3a and c, *P* < 0.001) in the SD + EE group. Co-localization analysis of LRP1 by IF assay showed that LRP1 was mainly expressed around vascular endothelial cells (Figure 3d and e). Compared with the Ctrl group, the expression of LRP1 was decreased in the prefrontal cortex (Figure 3d and f, *P* < 0.001) and hippocampus (Figure 3e and g, *P* < 0.001) in the SD group and increased in the prefrontal cortex (Figure 3d and f, *P* < 0.001) and hippocampus (Figure 3e and g, *P* < 0.001) in the EE group. Compared with the SD group, LRP1 expression was increased in the prefrontal cortex (Figure 3d and f, *P* < 0.001) and hippocampus (Figure 3e and g, *P* < 0.001) in the SD + EE group.

### 3.4 Effects of EE on RAGE expression in the prefrontal cortex and hippocampus in chronic SD

**Figure 4:**
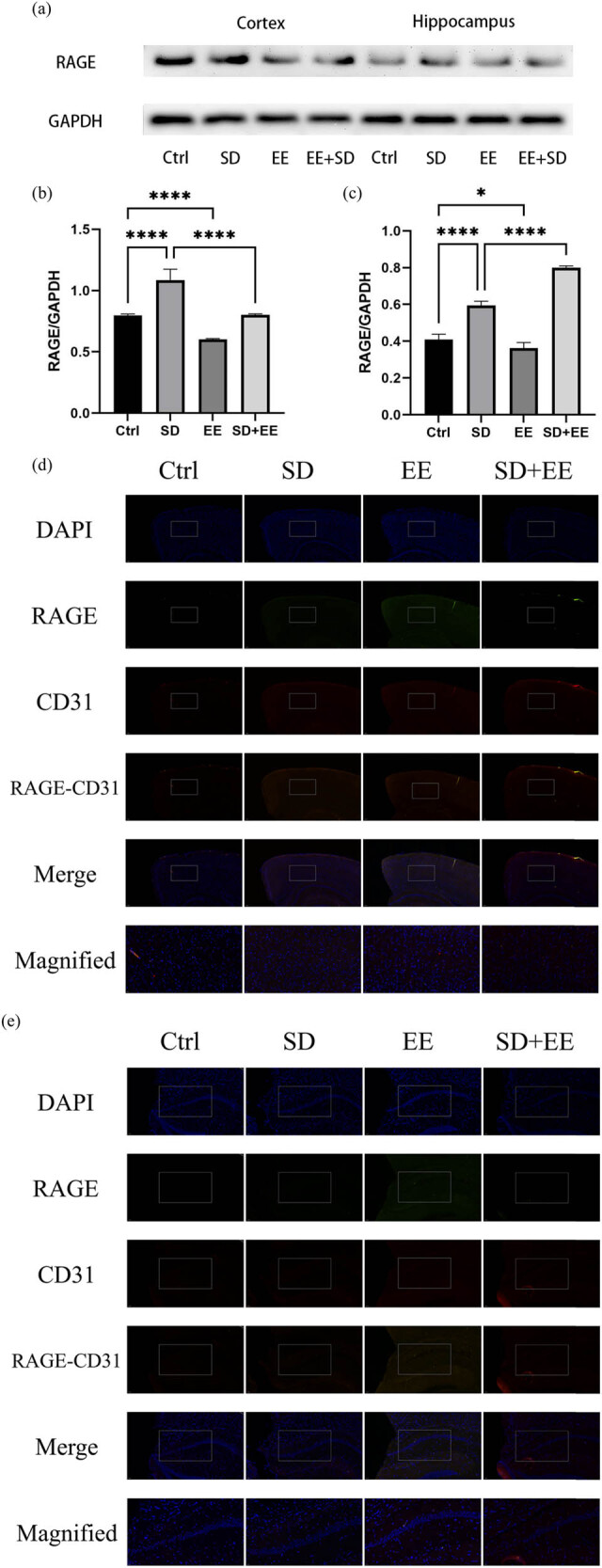

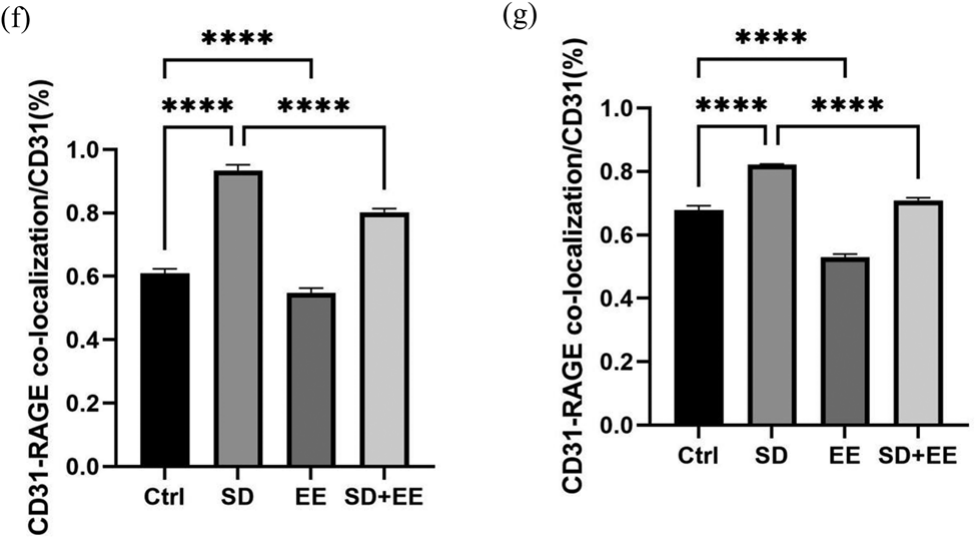
Effect of EE on RAGE expression in chronic sleep-deprived mice. Note: (a) prefrontal cortex and hippocampal RAGE protein bands in each group; (b and c) analysis of grayscale values of prefrontal cortex and hippocampal RAGE/GAPDH in each group; (d) immunofluorescence staining of prefrontal cortex RAGE (green), CD31 (red), and DAPI (blue) in each group, magnification scale bar = 100 μm, other scale bar = 20 μm; (e) immunofluorescence staining of prefrontal cortex and hippocampal RAGE (red), CD31 (green), and DAPI (blue) in each group, magnification scale bar = 50 μm, other scale bar = 20 μm; (f) percentage of CD31-RAGE co-localization area of prefrontal cortex stretching CD31 area in each group; and (g) percentage of CD31-RAGE co-localization area of hippocampal stretching CD31 area in each group (*n* = 3, ****P < 0.001).

RAGE is also a receptor expressed in the endothelium of brain microvessels. Its main function is to transport Aβ in the blood across the BBB to brain tissue [29]. Western blot analysis of RAGE showed that compared with the Ctrl group, the expression of RAGE protein was increased in the prefrontal cortex (Figure 4a and b, *P* < 0.001) and hippocampus (Figure 4a and c, *P* < 0.001) in the SD group, and the expression of RAGE protein was decreased in the prefrontal cortex (Figure 4a and b, *P* < 0.001) and hippocampus (Figure 4a and c, *P* < 0.01) in the EE group. Compared with the SD group, the expression of RAGE protein was decreased in the prefrontal cortex (Figure 4a and b, *P* < 0.001) and hippocampus (Figure 4a and c, *P* < 0.001) in the SD + EE group. Co-localization analysis of RAGE by IF assay showed that RAGE was mainly expressed around vascular endothelial cells (Figure 4d and e). Compared with the Ctrl group, the expression of RAGE was increased in the prefrontal cortex (Figure 4d and f, *P* < 0.001) and hippocampus (Figure 4e and g, *P* < 0.001) in the SD group, and the expression of RAGE was decreased in the prefrontal cortex (Figure 4d and f, *P* < 0.001) and in the hippocampus (Figure 4e and g, *P* < 0.01) in the EE group. Compared with the SD group, the expression of RAGE was reduced in the prefrontal cortex (Figure 4d and f, *P* < 0.001) and hippocampus (Figure 4e and g, *P* < 0.001) in the SD + EE group.

## 4 Discussion

Sleep plays an important role in maintaining neuronal circuits and signaling, and sleep disorders disrupt circadian rhythm and negatively affect the brain function and behavioral performance [30]. Current treatment modalities for sleep disorders are mainly drug-based, e.g., melatonin [31], chronic caffeine intake [32], and acute nicotine treatment [33], have been shown to alleviate SD-induced learning and memory deficits. Numerous studies have reported that EE improves neurogenesis and cognitive function [34,35]. Although there is a series of studies confirming the effectiveness of exercise in reducing Aβ accumulation; however, less research has been done on the EE. To date, it remains unclear how the EE reduces Aβ deposition.

On this basis, we investigated the effect of EE on cognitive impairment induced by chronic SD exposure and further dissected its mechanism of action. We found that EE could effectively ameliorate the adverse effects of chronic SD on cognitive function. EE could reduce Aβ deposition in chronic SD-exposed mice, through possible mechanism of modulating the expressions of Aβ transport-related proteins LRP1 and RAGE. EE could elevate the expression of LRP1 and decrease the expression of RAGE, which coordinated together to reduce Aβ accumulation.

EE is a complex of social, cognitive, and motor stimuli [36]. Compared to standard environments, EE provides experimental animals with more living space, more sensory stimulation, more possibilities for social interaction, and more opportunities for learning [37]. We designed the EE component to include both environmental (toys, balls, and swings) and voluntary movement (running wheels, bridges, and mazes). Both components may contribute to the beneficial effects of EE on cognitive function. Y-maze results showed that EE improved the reduced alternation rate due to SD with no statistically significant alteration in motor ability. Similarly, the novel object recognition test showed that EE improved the impaired recognitive memory caused by SD, which was consistent with the Y-maze results. These findings suggest that the EE improves learning, memory, and object recognition in chronic sleep-deprived mice. However, cognitive function consists of multiple domains such as executive function, attention, abstraction, and orientation [38]. Therefore, further experiments are needed to be performed to investigate the effects of EE on chronic SD in other cognitive domains.

There are few studies on the treatment of SD with EE, and the efficacy and possible mechanisms need to be further investigated. Gao et al. [39] found that an EE administration improved postoperative SD-induced memory deficits in rats, and the possible mechanism was that EE increased BDNF levels and decreased NT-3 levels in sleep-deprived rats, which may be mediated by attenuating changes in AMPAR subunit expression and decreasing GABAA receptor α1 subunit expression. Ghaheri et al. [40] indicated that adolescent residence in EE reduces SD-induced cognitive impairment and that elevated hippocampal BDNF levels are a possible mechanism for attenuating cognitive performance in sleep-deprived rats. These studies provide a partial theoretical basis for the idea that an EE can improve SD-induced cognitive impairment. Moreover, numerous studies have shown that EE can attenuate Aβ deposition in the brain of mice [41,42]. In addition, some studies have found that chronic SD may lead to Aβ deposition in the brains of wild-type rats or mice [27,43]. On this basis, whether the EE can have an effect on Aβ deposition in chronic sleep-deprived mice was investigated in the present study. Since Aβ1-42 is more toxic *in vivo* as it is more likely to form β folds and thus deposit in the brain [44]. Therefore, we analyzed the expression of Aβ1-42 in the prefrontal cortex and hippocampus of each group mainly by IF staining. Our results showed that Aβ1-42 was differentially elevated in the prefrontal cortex and hippocampus of chronic sleep-deprived mice, while its expression was reduced in mice with EE exposure.

The BBB is closely related to the balance of Aβ production and clearance in the brain [45]. The BBB has a selective filtration effect that stabilizes brain metabolism. BBB dysfunction can lead to impaired transport of Aβ from the cerebral to the peripheral circulation [46]. LRP1 and RAGE are the main transport proteins on BBB responsible for the transport and clearance of Aβ [47,48]. Donahue et al. [23] demonstrated that AD is closely associated with changes in the endothelial LRP1 and RAGE receptors in the BBB of human brain tissue. In particular, reduced LRP1 levels and increased RAGE levels in the BBB may lead to the failure of Aβ transport [49]. Studies have confirmed that physical exercise reduces amyloid plaque load in the brain through differential modulation of RAGE and LRP1, thereby ameliorating several pathological mechanisms of AD, such as neurovascular unit dysfunction or cognitive deficits [50,51]. However, in 2018, Ramos-Cejudo et al. found no significant alterations in LRP1, although they did find a reduction in RAGE in the hippocampus after prolonged treadmill exercise, which led to reduced Aβ deposition, but still mainly through inhibition of the APP amyloid pathway [52]. EE is similar to that of exercise therapy and may have an effect on LRP1 and RAGE. In our present study, our data confirmed that chronic SD leads to the reduced LRP1 expression in both prefrontal cortex and hippocampus and an elevated RAGE expression, impairing Aβ exchange between brain tissue and blood circulation, resulting in Aβ deposition in mouse brain tissue. In contrast, EE intervention ameliorated these adverse effects, resulting in the upregulation of LRP1 and downregulation of RAGE expression, and facilitating the transport of Aβ across the BBB to the outside of the brain.

Worth noting, EE may play a multifactorial role in ameliorating the various pathophysiological mechanisms associated with SD-induced cognitive impairment and neurodegeneration, such as tau and Aβ aggregation, inflammation, oxidative stress, cellular scorching, and endothelial dysfunction [53]. Our study only deals with Aβ clearance, while other factors mentioned above may also be involved. Our study of Aβ metabolism mainly focused on the transport related LRP1 and RAGE. Further experiments should be performed to evaluate other metabolic pathways of Aβ.

## 5 Conclusion

In conclusion, our experiments demonstrate that EE is effective in ameliorating the adverse effects of chronic SD on cognitive impairment and Aβ deposition, through coordinating the expression of Aβ transport-related proteins LRP1 and RAGE across the BBB. Our data provide evidence for the development of EE as potential therapy against sleep disorder, although further experiments are required to investigate other essential mechanisms underlying.
